# Treatment of sugar processing industry effluent up to remittance limits: Suitability of hybrid electrode for electrochemical reactor

**DOI:** 10.1016/j.mex.2017.05.001

**Published:** 2017-05-10

**Authors:** Omprakash Sahu

**Affiliations:** School of Chemical, Food Engineering, Bahir Dar Institute of Technology, Ethiopia

**Keywords:** Electrochemical method for industrial wastewater treatment, Anode material, Electrocoagulation, Filtrations, Physicochemical analysis, Sedimentation

## Abstract

Sugar industry is an oldest accommodates industry in the world. It required and discharges a large amount of water for processing. Removal of chemical oxygen demand and color through the electrochemical process including hybrid iron and aluminum electrode was examined for the treatment of cane-based sugar industry wastewater. Most favorable condition at pH 6.5, inter-electrode gap 20 mm, current density 156 A m^−2^, electrolyte concentration 0.5 M and reaction time 120 min, 90% COD and 93.5% color removal was achieved. The sludge generated after treatment has less organic contain, which can be used as manure in agricultural crops. Overall the electrocoagulation was found to be reliable, efficient and economically fit to treat the sugar industry wastewater.

• Electrocoagulation method for sugar processing industry wastewater treatment Optimization of operating parameters for maximum efficiency.
• Physicochemical analysis of sludge and scum.
• Significance of hydride metal electrode for pollutant removal.

## Graphical abstract

**Figure:**
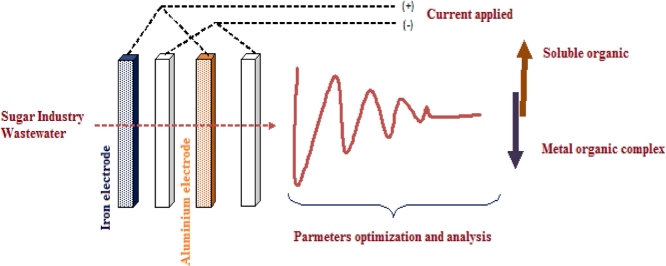

## Method details

### Overview

Electrocoagulation is advance technique being employed in the treatment of water and watewater [1]. This methds offer an alternative to the use of metal salts or polymers and polyelectrolyte addition for breaking stable emulsions and suspensions. The destabilization mechanism occurred, in compression of double layer; charge neturalization and flock formation [2], [3]. In considering being several technology electrocoagulation treatments is adequate and survivable one. Electrochemical treatment also showed better achievements for removal of suspended colloidal from water and wastewater as compared to classical coagulation treatment process [4]. Different combinations of the parameter are responsible for performance electrochemical process; in one of them is electrode material. The suitable electrode material like iron [5], aluminum [6] and other metals like carbon, mild steel, and stainless steel [7], including combination of iron and aluminium [8] are used for treatment of different industrial waste water. Electrochemical treatment processes have a simple arrangement and portable for transportation, including convenient to handle.

The sugar industry is coming under prime agro- business. To process one ton of sugarcane large quantity of fresh water required and large amount of wastewater release as wastewater. The effluent generated from sugar processing industry has miscellaneous quality like contains high BOD, COD, grease, dissolved solid etc. To comply with the environmental norms for the release of effluent, generally conventional (settling, filtration and pond treatment) methods were brought in practices for the economical purpose [9]. To minimise the contaminated level from wastewater an attempted has been made with electrocogulation method. In present work, electrochemical methodology has been applied for the treatment of sugar industry wastewater with hydride (iron and aluminum) electrode. The quality of wastewater was controlled by operating parameters of electrochemical reactor and brings up to acceptable norm. The scum and sludge were analyzed by thermal degradation and energy x-ray diffraction methodology.

## Methodological protocols

### Material

Aluminum (Al-8011) and the iron (SS-302) sheet were purchased from local market. Measure dimension of the sheet was cute with a metal cutter and clean before used. Laboratory grade chemical was used without purification. The waste water preserved in 4 °C deep freeze untilled used and composition of effluent is presented in Table 1.

**Table 1:** Characteristic of SIWW before treatment.

S.No	Characteristics	Parameters
1	Color	Dark Yellow
2	pH	5.5
3	COD	3682 mg/l
5	Phosphate	5.9 mg/l
6	Protein	43 mg/l
7	Total solid	1287 mg/l
8	Suspended solid	340 mg/l
9	Dissolved solid	947 mg/l
10	Chloride	50 mg/l
11	Hardness	900 mg/l

### Experimental methods

**Fig. 1:**
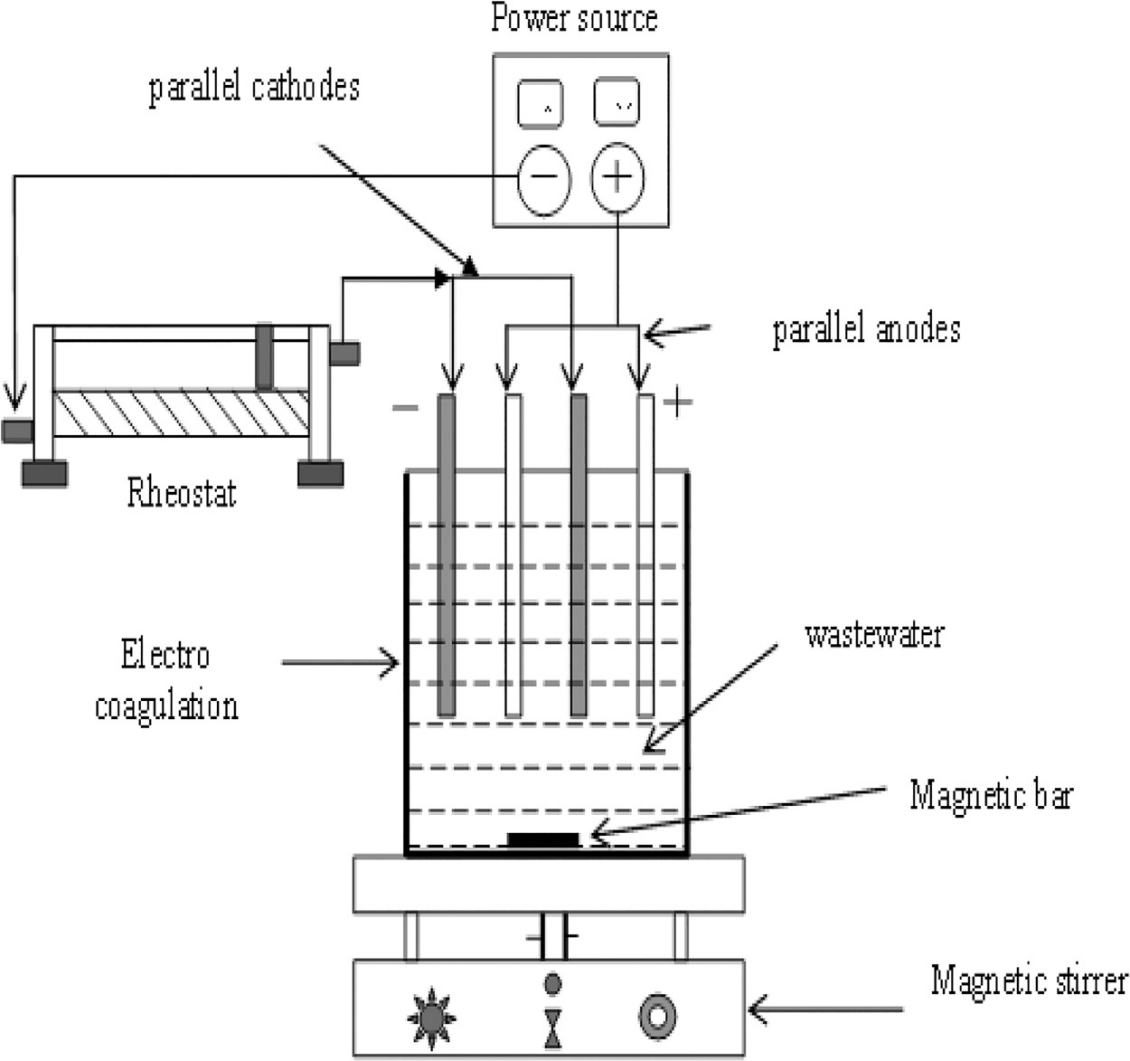
Experiment setup of electrocoagulation.

The complete experimental is shown in Fig. 1. The manageable electrochemical container was prepared from transparent glass and fitted with a pair of anode and cathode. These electrodes were arranged in parallel and maintained 20 mm space between them. A DC power was supply through electrodes. The current supply and voltage were measured with ammeter and voltmeter. The electrochemical reactor was used for batch and continuous experiments. The configuration of the reactor is given in Table 2. When a potential (usually of direct current) is applied from an external power source, the anode material undergoes oxidation, while the cathode will be subjected to reduction or reductive deposition of elemental metals. The electrochemical reactions with metal M as anode may be summarized as follows:

**Table 2:** Characteristics of electrochemical reactor.

S.No		
Electrode		
1	Material(anode and Cathode)	Iron-Aluminium
2	Shape	Rectangular (square)
3	Size	8 cm × 8 cm
4	Thickness	2 mm
5	Plate arrangement	Parallel
6	Effective electrode surface area	64 cm^2^

Reactor Characteristics		
1	Make	Perspex glass
2	Reactor type	Batch mode
3	Dimensions (cm)	10.7 × 10.7 × 13.7
4	Volume (dm^3^)	1.5
5	Electrode gap	2 cm
6	Stirring mechanism	Magnetic bar

Power Supply		
1	Voltage range	0–30 V
2	Current	0–5 A

**At the anode:At the anode:**

(1) M_(s)_ → M_(aq)_*^n^*^+^ + *n*e^−^

(2) 2H_2_O_(l)_ → 4H_(aq)_ + O_2(g)_ +4e^−^

**At the cathode:**

(3) M_(aq)_*^n^*^+^ + *n*e^−^ → M_(s)_

(4) 2H_2_O_(l)_ + 2e^−^ → 2H_2(l)_ + 2OH^−^_(aq)_

## Analytical methods

The COD of the samples was determined by the standard dichromate reflux method [10]. The chloride concentration was determined by the standard titrametric Volhard method [11]. Sulphates and the phosphates were determined by using standard methods [10]. The concentrations of the metal ions in the filtrate and the residue were determined by using an atomic absorption spectrometer (GBC, Model Awanta, Australia). The protein content was determined by the Bradford method [12]. The colour of the sample was measured in terms of the absorbance at *λ* = 475 nm using a UV–vis spectrophotometer (Model Lambda 35) from Perkin-Elmer Instruments, Switzerland. Thermal analysis (TGA/DTGA/DTA) of the sludge obtained after the treatment of SIWW by using a TG analyser (Pyris Diamond, Perkin-Elmer).

## Method validation

### Quality control with initial pH

**Fig. 2:**
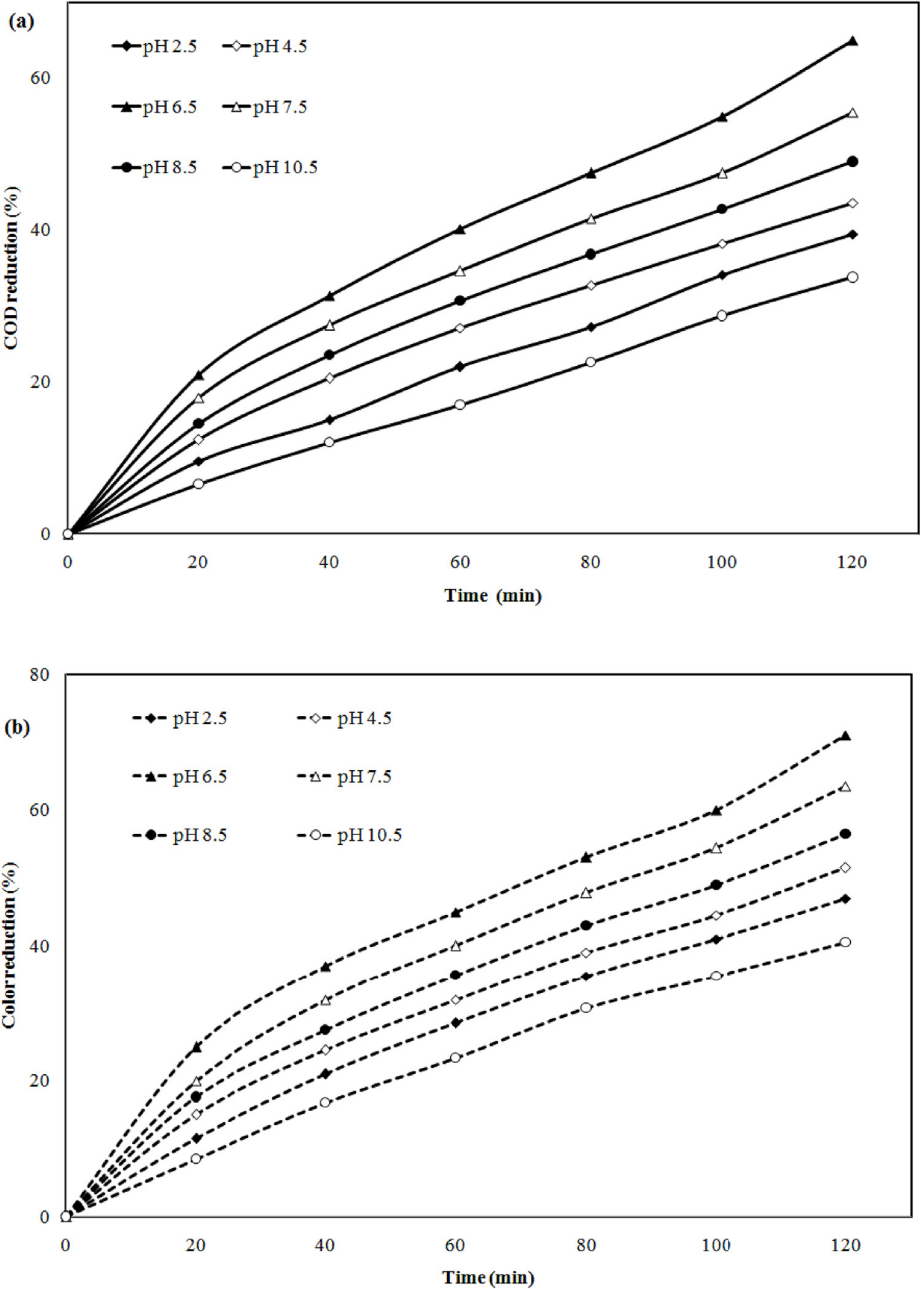
Effect of pH on (a) COD and (b) color reduction at CD = 78 A m^−2^, ED = 20 mm, pHi = 5.5, CODi = 3682 mg/l, Color = 350PCU and time (t) = 120 min.

The performance of electrocoagulation treatment depends on the nature of water and wastewater (acid or alkaline). To examine the effect of pH, initial pH was varies from pH 2.5–10.5 at 3682 mg/l COD, 350 PCU color, 78 A m^−2^current density (CD), 20 mm electrode distance (ED) and 120 min time (t) respectively. The removal efficiency by 4 plate configuration, hydride electrode (HE) iron and aluminum is represented in Fig. 2(a) and (b). During the experiment, it has been observed that COD 35.5%, 43.6% and color reduction 47%, 51.5% increase with an increase in pH from pH 2.5 to pH 4.5. At pH 6.5 highest 65% COD and 71% color removal was achieved. Further increase in pH 7.5, 8.5, 10.5 reductions of COD 55.5%, 49%, 33.8% and color 63.5, 56.5, 40.5% were decreases. The increasing and decreasing of reduction efficiency may be due to the formation of hydroxide and metal cations of electrode used [13]. To treat the peat bog drainage synthetic wastewater authors has been used Al/Fe (anode/cathode) electrode material and found 90% COD and 80% DOC at pH4 (high acid range) current density 100 A m^−2^ and treatment time 10 min. But present experimental shows 65% COD reduction at pH 6.5 which is near to allowable limit [14].

**Fig. 3:**
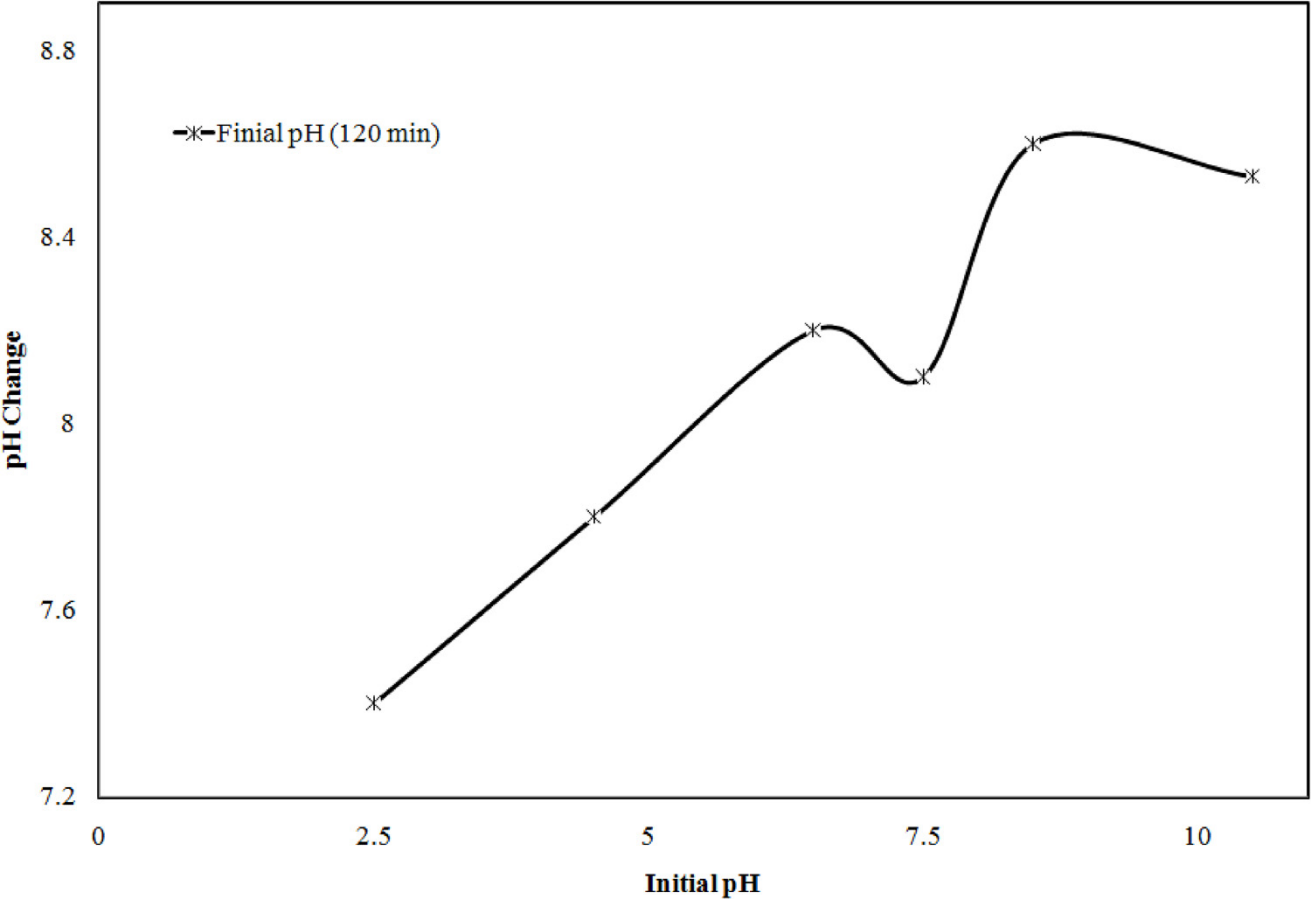
Effect of initial pH on pH change at 120 min reaction time.

During the electrolysis process at initial pH, change in pH with respect to time (120 min) was also observed, which is shown in Fig. 3. It can be seen that the pH was increased with increase with time for pH 2.5, 4.5, 6.5, 7.5, 8.5, 10.5 to 8 was found pH 7.4, 7.8, 8.2, 8.1 and 8.5 and then it decreased for pH 10 was pH 8.53. At high acidic nature of sample metal, ion formed hydroxide ions, which is consumed by the acid of solution and at high basic nature of sample hydroxyl ions generated at the cathode, which lead to decrease the pH of sample.

**Fig. 4:**
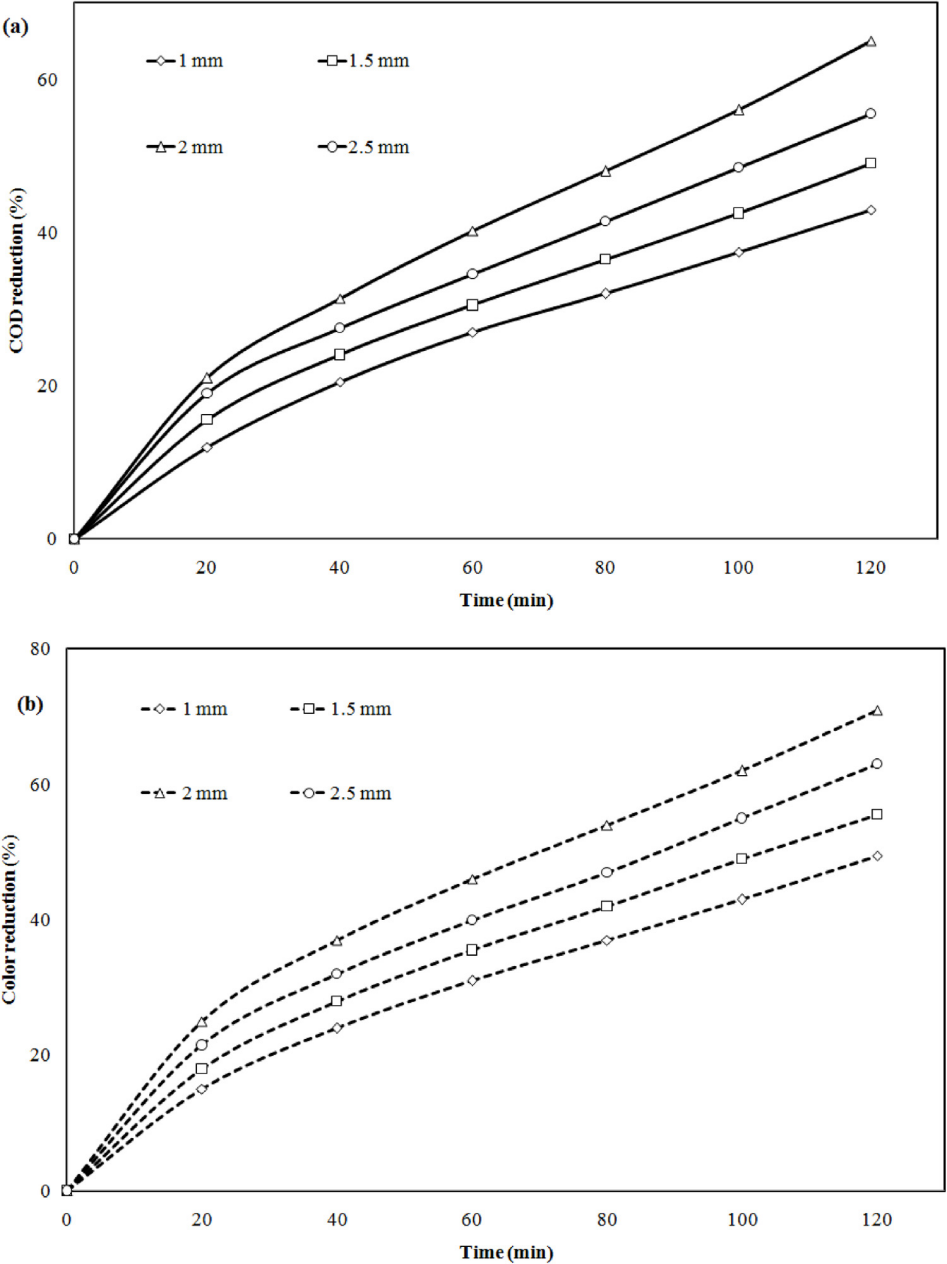
Effect of electrode distance on (a) COD and (b) color reduction at CD = 78 A m^−2^, pH = 6.5, CODi = 3682 mg/l, Color = 350PCU and time (t) = 120 min.

To investigate the effect of the gap between two electrode an experiment was carried out at 10 mm to 25 mm, pH 6.5, CD 78Am^−2^, 120 min with initial COD and color. It was observed that increasing in electrode distance the chemical oxygen demand (COD) and color removal were accelerate the time, which is shown in Fig. 4(a) and (b). Initially when distance between electrodes were maintained 10 mm, 15 mm, 20 mm, the COD 43%, 49%, 65% and color reduction was 49.5%, 55%, 71% respectively. Repeatedly increase the gap 25 mm the COD 55.5% and the color reduction was reduced to 63%. This may be due to the increases in internal resistance between the electrodes at a same current density that leads to the decrease of optimum ion production [15].

## Quality control with current density

**Fig. 5:**
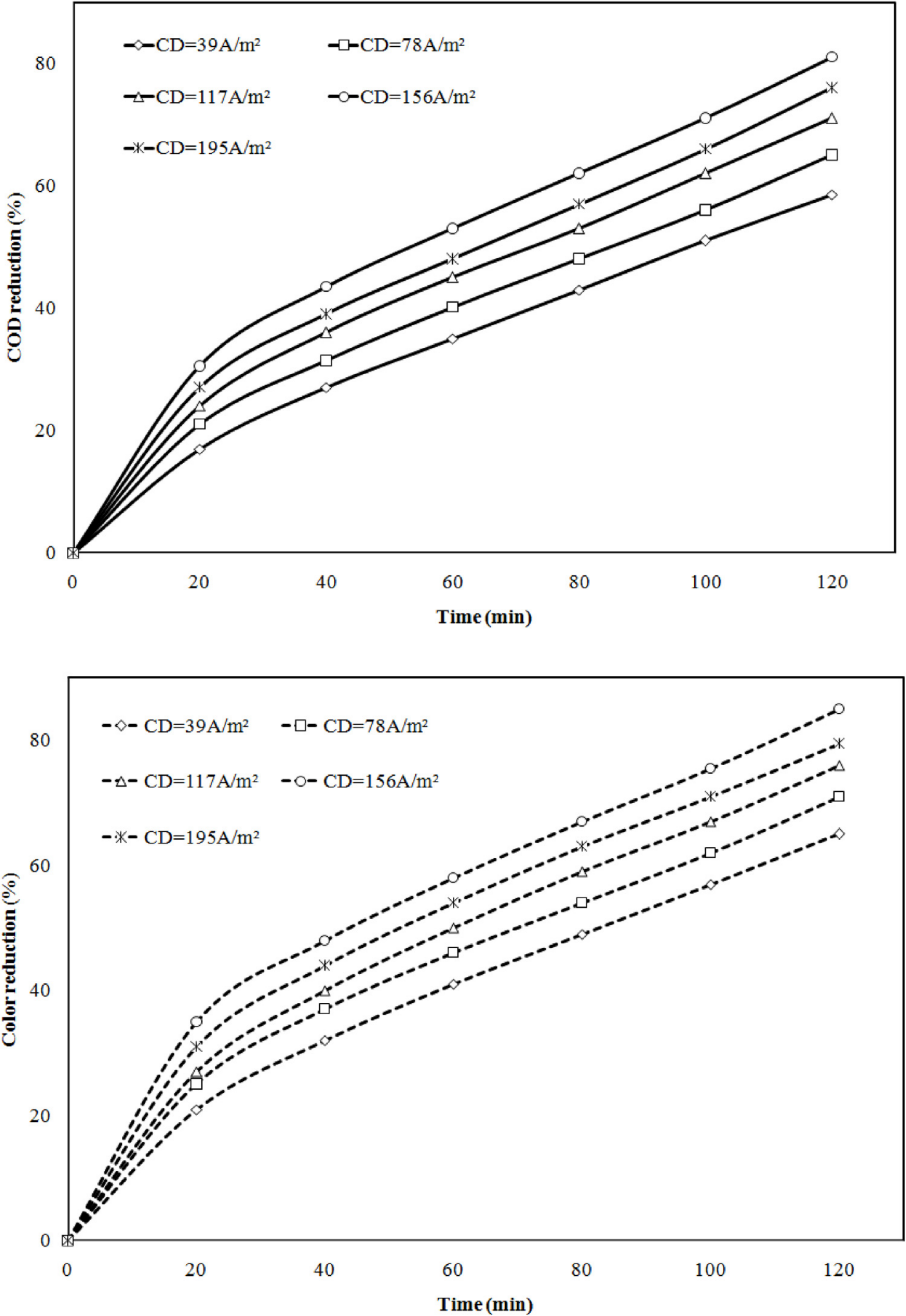
Effect of current density on (a) COD and (b) color reduction at ED = 20 mm, pH = 6.5, CODi = 3682 mg/l, Color = 350PCU and time (t) = 120 min.

The influence of current density (39 A m^−2^ to 195 A m^−2^) on the elimination of chemical oxygen demand (COD) and color has been studied at optimum pH. The results represent in Fig. 5(a) and (b). It has been observed that the removal efficiency was rising with current (1 A to 4 A) supply to electrode up to certain limit, further supply (5 A) drop the efficiency. From the result, ultimate achievement was 81% COD and 85% color removal at 156 A m^−2^ current density. At maximal current density 195 A m^−2^ it fell down to 76% COD and 79.5% color removal. When current density was 39 A m^−2^, 78 A m^−2^ and 117 A m^−2^ the COD 58.5%, 65%, 71% and color removal 65%, 71% was observed. It is accepted that with increase in current density ion production increase (Faraday’s Law), which is sufficient to neutralize the colloidal present in wastewater, further addition of current density brings destabilization due to overdosing [16].

**Fig. 6:**
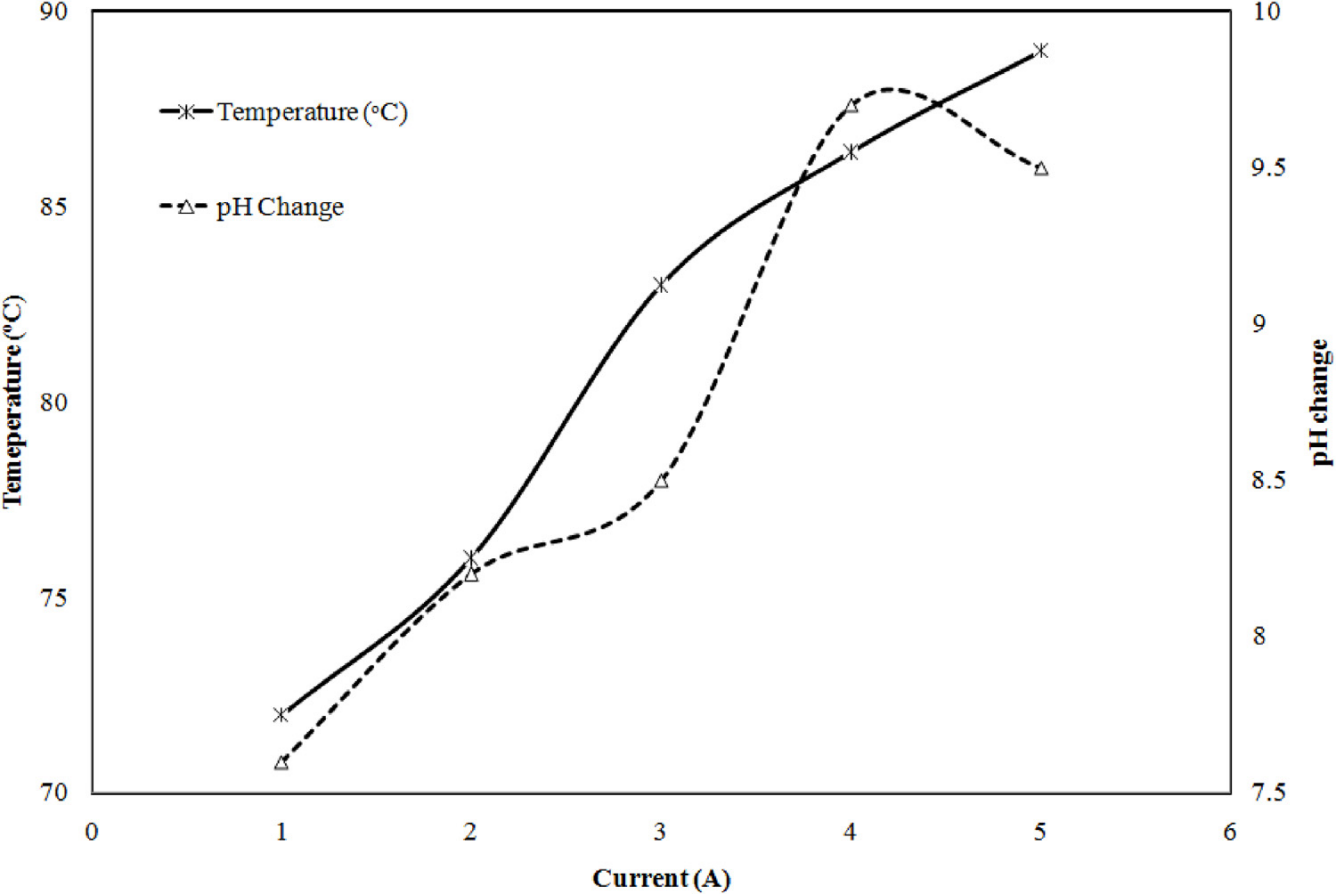
Effect of current density on pH change and temperature at 120 min.

In electrochemical process current density also affect the initial pH and temperature of the sample with time. Probably it may be ions released from the electrodes. When current density was 39, 78, 117, 156 and 195 A m^−2^ the pH 7.6, 8.2, 8.5, 9.7 and 9.5 at optimum pH was observed. The change in pH and temperature with current density is shown in Fig. 6. Similarly when current density was 39, 78, 117, 156 and 195 A m^−2^ the temperature change 72, 76, 83, 86.4 and 89 °C was found at 120 min of reaction time. This phenomenon indicates the formation of H^+^ ions speed up with anodic dissolution of the electrode, which results, change in chemical composition of wastewater to eliminate the pollution and decrease the pH of wastewater. In same manner when boron containing wastewater treated with electrocoagulation, temperature increase from 19.85 to 59.85 °C, with respect to removal efficiency from 84% to 96% [17].

**Fig. 7:**
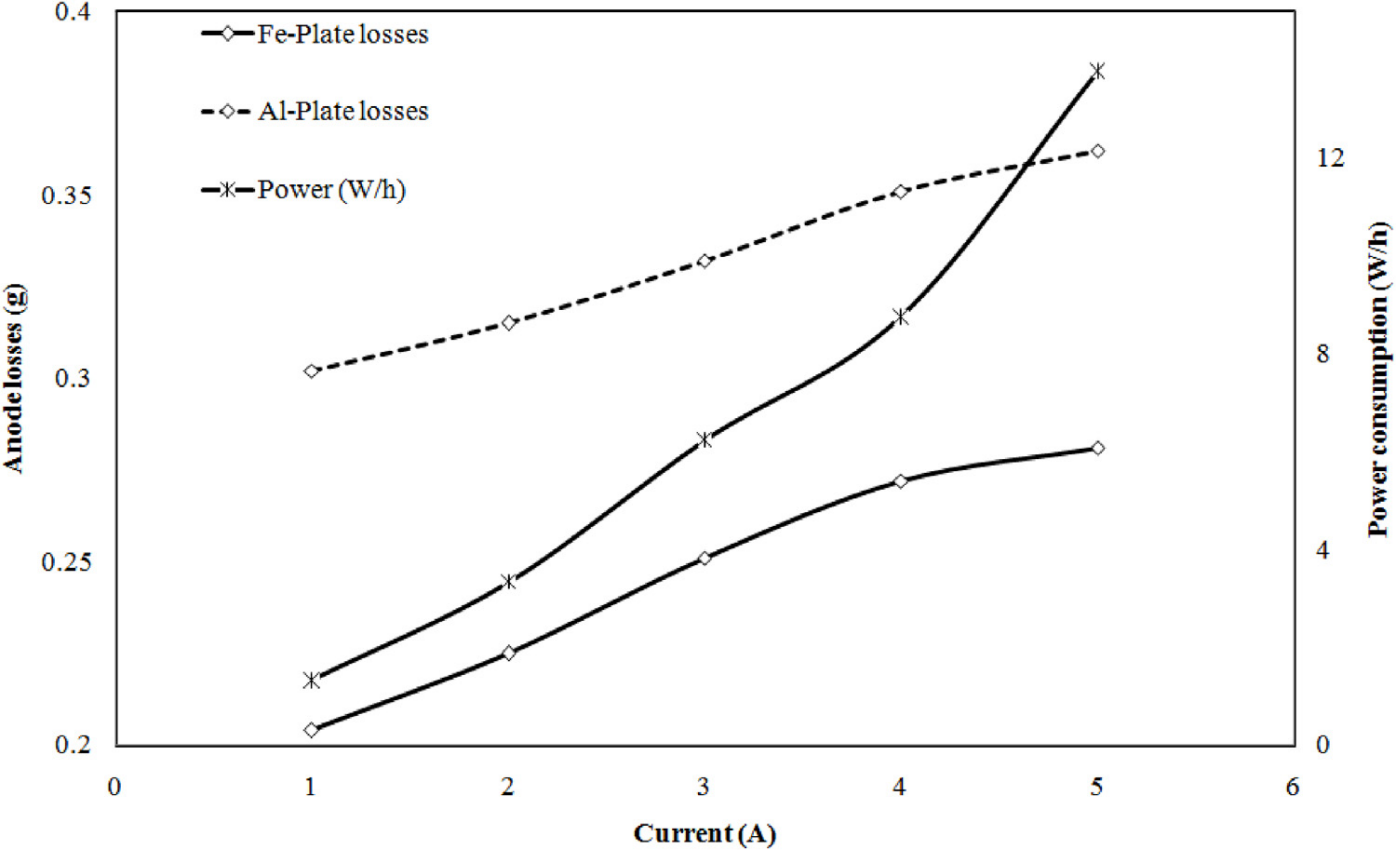
Effect of current density on anode consumption and power consumption.

The effect of applied current 1 A–5 A on power consumption and anode losses was carried out at optimum pH (pH 6.5) is shown in Fig. 7. The result shows the addition of current increase the power and anode losses. When current density was 39, 78, 117, 156 and 195 A m^−2^, the power 1.32, 3.34, 6.23, 8.75, 13.75 kW/h the iron electrode losses 0.204, 0.225, 0.251, 0.272, 0.281 g and aluminum anode losses 0.302, 0.315, 0.332, 0.351, 0.361 g was observed. During the treatment, it was also found that aluminum electrode more dissolves as compared to the iron electrode. It may be due to the electrochemical property of aluminum [18].

**Fig. 8:**
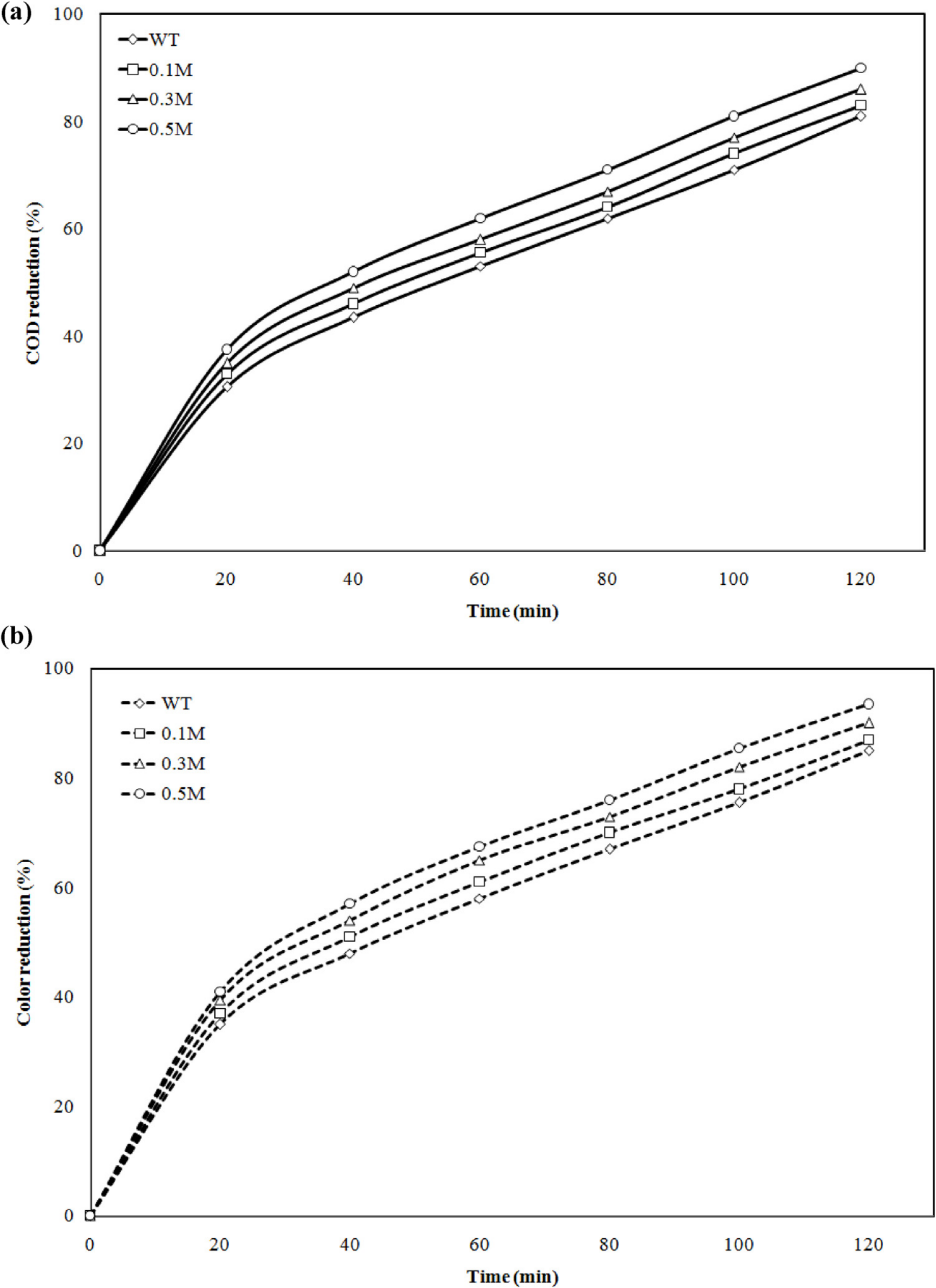
Effect of electrolyte concentration on (a) COD and (b) color reduction at ED = 156 A m^−2^, ED = 20 mm and t = 120 min.

The effect of the electrolyte was carried out from 0.1 M, 0.3 M and 0.5 M concentration of NaCl (sodium chloride) shown in Fig. 8(a) and (b). During the electrochemical process addition of electrolyte NaCl 0.1 M, 0.3 M, 0.5 M increase the COD 83%, 86%, 90% and color 87%, 90.1%, 93.5% removal efficiency of process. Without electrolyte, the removal efficiency was 81% COD and 85% color in optimum condition. This attributes to more amount of hypochlorite ion were generated in the sample, which alters the nature of organic present in the effluent. In literature there are different types of an electrolyte like NaCl, BaCl_2_, KCl, Na_2_SO_4_ and KI has been used for industrial waste water treatment. Kuokkanen et al. used NaCl as electrolyte and attended 96% phosphorus from synthetic water and 93% from dairy industry waste by using hybrid electrode (Al and Fe) [14].

## Analysis of scum and sludge

**Fig. 9:**
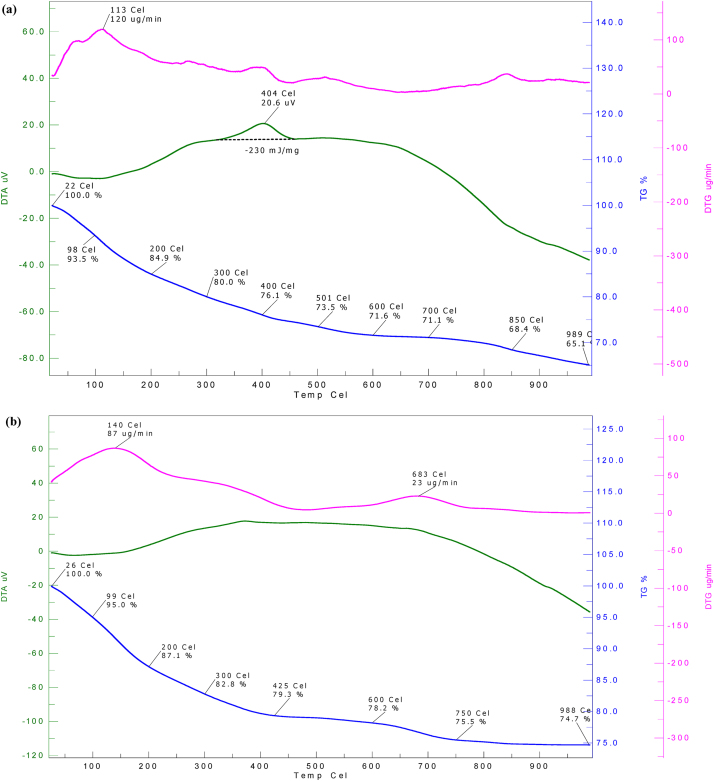
Thermal analysis (DTG, DTA and TG) of (a) scum and (b) sludge.

The thermogravitmetry analysis (TGA), differential thermogravitmetry analysis (DTGA) and derivative thermal analysis (DTA) of scrum sludge and supernaent dried mass thermal characteristics was studied. Fig. 9 (a) and (b) shows DTG, DTA, and TG curve of the scum and precipitated sludge obtained at pH 6.5. TG curve shows that up to 300 °C temperature, dehydration and volatilization (reduction of volatiles) of the sample takes place and a reduction of 20 μg wt% and 17.2 μg wt% is recorded for scum and sludge respectively. From 300 °C to 600 °C, in the span of 300 °C, the precipitate is oxidized, losing about 8.4 wt% of scum and 4.6 wt% of the sludge. After 600 °C the oxidation is slow and the final decrease in weight of the scum and sludge is recorded as 6.5 wt% and 3.5 wt% respectively. The ash in scum and sludge remains 65.1% and 74.7%. The DTG curve shows the peak rate of weight loss of 87 μg min^−1^ at 140^ °^C for sludge and 120 μg min^−1^ at 113^ °^C for scum. The oxidation of top scum is exothermic, with a heat evolution of 230 MJ kg^−1^ at 400 ^°^C. This study shows the inorganic nature of the sludge.

**Fig. 10:**
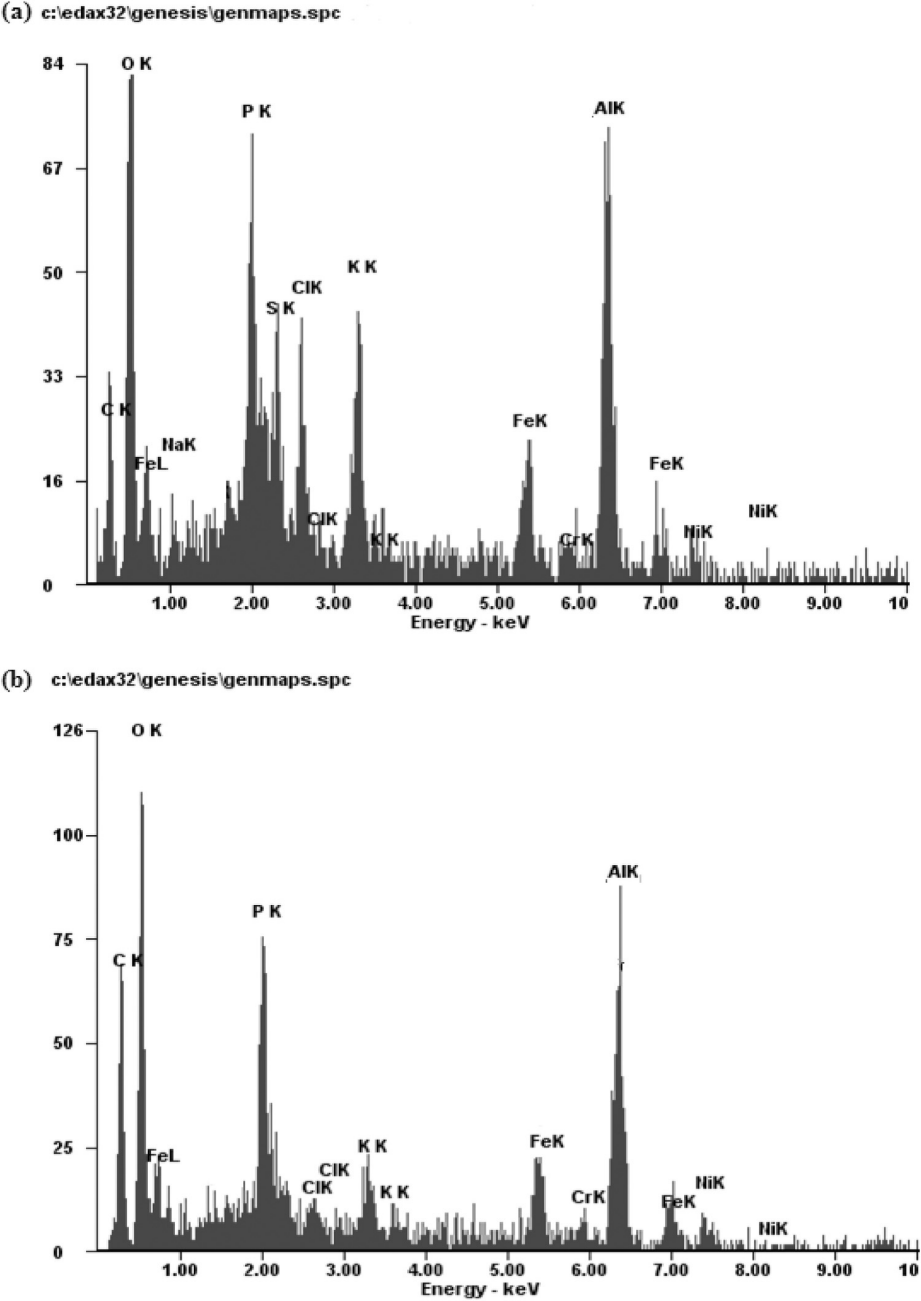
Energy-dispersive X-ray spectroscopy analysis of (a) scum (b) sludge.

The EDAX analysis of sum and sludge is shows in Fig. 10 (a) and (b). The result indicates the presence of different element C, O, K, P, Cr, Al, Fe, Ni and Cl. The Fe/Al generated from the electrode, comes down in the sludge, due to this it FeK and AlK in sludge is 16.82 wt% and 25.41 wt%. Carbon also gets deposited in the sludge and the CK value is 32.27 wt%. FeK and AlK in scum were 15.19 wt% and 20.35 wt%, while CK 23.30 wt% .After incineration, the ash obtained can be used as a micronutrient for blending it either with organic manure or mixed with raw materials to manufacture bricks. The scum contains C, O, K, Fe and Al in higher proportions along with small amounts of Na, S, Cl, Cr and Ni. The scum can also be used as a micronutrient after incineration.

## Outcomes

Treatment of sugar industry wastewater by an electrochemical process with hybrid electrode was found very suitable for the removal of various pollutants. It shows the high capability to treat the highly contaminated wastewater up to reprocessing stages. The excellent achievement was 90% chemical oxygen demand and 93.5% color reduction at pH 6.5, electrode distance 20 mm, current density 156Am^−2^, electrolyte concentration 0.5 M and treatment time 120 min respectively. The sludge and slurry generated after electrocoagulation with iron and aluminum electrodes are suitable for agricultural purpose Best removal efficiency was achieved in the pH 6.5. At optimum pH, 65% chemical oxygen demand (COD) and 71% color removal were attended at a current density of 78 A m^−2^, an internal electrode distance of 20 mm and operating time of 120 min. The energy consumption was 8.75 kW/h with anode losses 0.272 g (iron) and 0.375 g (aluminum) at 156 A m^−2^ current density. Finally, electrocoagulation treatment with the hybrid electrode is a safe to operate, economical, convenient and adequate process for pollutant elimination.
